# Optimization of Functional Gluten‐Free Cake Formulation Using Rice Flour, Coconut Flour, and Xanthan Gum via D‐Optimal Mixture Design

**DOI:** 10.1002/fsn3.4523

**Published:** 2024-11-25

**Authors:** Aidin Taromsari, Babak Ghiassi Tarzi

**Affiliations:** ^1^ Department of Food Science and Technology, Science and Research Branch Islamic Azad University Tehran Iran

**Keywords:** dietary fiber, functional food, gluten sensitivity, hydrocolloid, quality enhancement, response surface methodology

## Abstract

Prolamins in wheat, barley, and rye cause celiac disease (CD), non‐celiac gluten sensitivity (NCGS), and wheat allergies (WA). Although rice can be a suitable alternative, gluten‐free rice flour products are less technologically advanced. However, coconut flour and xanthan gum could enhance product quality, fiber content, and functional properties. This study employed a response surface D‐optimal mixture design to investigate the impact of substituting Sadri Hashemi rice flour (RF) (15.60%–31.19%) with low‐fat desiccated coconut flour (CF) (0%–15.59%) and xanthan gum (XG) (0%–0.31%) in 17 runs based on batter weight. Eleven responses were analyzed and exhibited significant and valid models (*p* < 0.0001), with satisfactory coefficient of determination (*R* ^2^ > 0.85) values and non‐significant lack of fit (*p* > 0.1). Substitution at all levels significantly increased batter viscosity (*R* ^2^ = 0.990) and specific weight (*R* ^2^ = 0.995). Differences in cake crumb color (Δ*E*) decreased when RF was substituted with CF, while maximum Δ*E* elevation was observed with 0.31% XG due to synergistic effects with 5.09% CF (*R* ^2^ = 0.974). Increasing the RF/XG ratio (0% CF) resulted in decreased water activity (*a*_*w*_), while increasing CF increased *a*_*w*_ synergistically (*R* ^2^ = 0.986). Moisture and texture hardness increased significantly with RF substitution up to 7.518% CF and 0.170% XG and 8.944% CF and 0.285% XG, respectively (*R* ^2^ = 0.976 and 0.993). The volume index decreased with CF and XG (*R* ^2^ = 0.989). Sensory acceptances were reduced by increasing XG while improved with optimal CF levels, predicted at 6.415% (*R* ^2^ = 0.997), 6.784% (*R* ^2^ = 0.999), 9.001% (*R* ^2^ = 0.999), and 5.924% (*R* ^2^ = 0.999) for color, taste, texture, and overall acceptance, respectively. Two‐sided prediction interval confirmation runs with 95% confidence (*p* ≤ 0.05) validated the models' accuracy and prediction of optimized combinations. The optimized gluten‐free cake formula (23.395% RF, 7.795% CF, 0% XG) achieved an 88.0% desirability score. Developing valid regression models facilitates a comprehensive investigation of cake quality and sensory properties, ensuring a viable gluten‐free product for individuals with gluten‐related disorders.

## Graphical abstract

Prolamins in wheat, barley, and rye cause celiac disease, non‐celiac gluten sensitivity, and wheat allergies. While rice is a suitable alternative, gluten‐free products made from rice flour are less technologically advanced. This study investigated the effects of substituting Sadri Hashemi rice flour (RF) with low‐fat desiccated coconut flour (CF) and xanthan gum (XG) in gluten‐free cake formulations using a D‐optimal mixture design. Eleven responses were analyzed, showing significant models (*p* < 0.0001) with satisfactory predicted coefficient of determination values. Substitution affected the batter and cakes' physicochemical and sensory properties, making 11 robust models for further predictions. The optimized gluten‐free cake (23.395% RF, 7.795% CF, 0% XG) achieved an 88% desirability score.

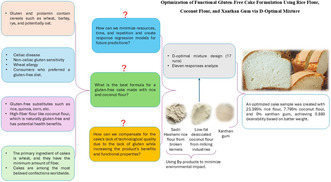

## 1 Introduction

Scientific advancements offer various treatments for overcoming illnesses. Notably, metabolic syndromes like obesity, diabetes, hypertension, and allergies are linked to both hereditary factors (like celiac disease) and dietary choices (Saklayen 2018; Stamnaes and Sollid 2015). These diseases highlight the powerful role of food in our health. Fortunately, advanced food science and technology are developing alternative food formulations that meet patients' needs and help prevent diet‐related diseases.

Gluten, a protein found in wheat, barley, and rye, contains gliadin (a member of the prolamin group) and glutenin and can stimulate immune responses in susceptible individuals. These responses are conditions such as CD, NCGS, and IgE‐mediated WA. Approximately 10% of the population experience some form of immune reaction to gluten or wheat (Cenni et al. 2023; Vasagar and Leonard 2014). CD, the most prevalent autoimmune disease affecting the small intestine, is triggered by a genetic condition inhibiting the metabolism of gluten's prolamin proteins (Lebwohl, Sanders, and Green 2018). CD also affects 1%–2% of the global population (Lundin and Wijmenga 2015). An increase in CD prevalence, particularly in Europe and the US, since the 1970s, with an estimated annual growth rate of 7.5% in recent decades, has been indicated (King et al. 2020; Vasagar and Leonard 2014). The highest CD prevalence is in Europe and Oceania, affecting around 0.8% of the population. Interestingly, females and children are more susceptible to CD than males and adults (Singh et al. 2018). CD can present symptoms beyond the digestive system and is often associated with other autoimmune disorders (Durazzo et al. 2022). Both CD and NCGS are connected to a range of diseases, including dermatitis herpetiformis, gluten ataxia, type 1 diabetes, autoimmune thyroid conditions, gastrointestinal dysbiosis, constipation, diarrhea, visceral hypersensitivity, metabolic rate dysfunction, peripheral immune communication and neuro‐immune communication dysfunctions, gluten neuropathy, gluten‐sensitive epilepsy, movement problems, gluten encephalopathy, myopathy, reduced energy expenditure, liver problems, malnutrition, and metabolic thermogenesis decreasing (Ch'ng, Jones, and Kingham 2007; Daulatzai 2015; Durazzo et al. 2022; Freire et al. 2016; Lebwohl, Sanders, and Green 2018; Polo et al. 2020; Serena et al. 2015; Zis and Hadjivassiliou 2019). Beyond wheat gluten's prolamin (gliadin), rye (secalin), barley (hordein), and potentially oats (avenin), grains like triticale (a wheat–rye hybrid), spelt, kamut, and einkorn may contain gluten‐like proteins that cause immune reactions in sensitive individuals (Biesiekierski 2017; Chan 2014; Rao and Hemalatha 2014; Rosell and Gómez 2014). As disease management, strict gluten‐free diets are crucial to reducing inflammations. Suitable replacements for prolamin‐containing grains will be rice, corn, sorghum, millet, teff, amaranth, buckwheat, quinoa, and wild rice (Siddiqui et al. 2022; Thompson 2009).

Rice (*Oryza sativa* L.) is a staple food for two‐thirds of the world's population. It is the cereal grain with the lowest protein and the highest lysine content. However, rice kernels lack vitamins A, C, and D and contain minimal fiber. Rice is naturally gluten‐free due to its low prolamin content, making it suitable for a gluten‐free diet. Its pleasant flavor, unique characteristics, white color, ease of digestion, hypoallergenic properties, and low sodium content make rice an excellent choice for extruded products, baked goods, confectionery items, and even infant formula (Arendt and Zannini 2013). Both polished and broken rice grains can be milled into flour, but it is more cost‐effective to produce rice flour from broken kernels. Broken rice may contain damaged starch granules, and milling without prior sorting can make it difficult to manufacture high‐quality products. Because of the low prolamin content, the protein network in rice flour is not strong enough to retain and expand with carbon dioxide produced by chemical or organic leavening agents. Instead of gluten, whey protein, eggs, and hydrocolloids like xanthan gum and hydroxypropyl methylcellulose (HPMC) can be used in rice‐based products (Arendt and Zannini 2013; de la Hera et al. 2013; Rosell and Gómez 2014).

More than 40 years ago, the concept of fiber consumption as a crucial factor in reducing the risk of chronic diseases such as cancer, cardiovascular disease (CVD), and type 2 diabetes emerged (Trinidad et al. 2006). Dietary fiber provides a wide range of health benefits, leading to recommendations to increase daily intake. However, despite these recommendations, several studies have shown that most people do not meet the suggested daily intake of 25–30 g of fiber (Osorio‐Diaz et al. 2014).

The coconut palm (*Cocos nucifera* L.), a member of the Arecaceae or Palmae family, is a monocotyledon with many applications. It is highly valued for its numerous benefits in nutrition, medicine, and cosmetics, consistently ranking among the top 10 plants. When coconut oil is extracted from the solid endosperm (kernel), the remaining white residue is referred to as virgin coconut meal (VCM). This valuable byproduct can be further processed into a high‐fiber flour, adding functional properties to various food products (Kaur et al. 2019). The popularity of coconut flour and fruit production is rising due to its potential health benefits, including its ability to reduce the risk of type 2 diabetes, CVD, and colon cancer (Trinidad et al. 2006). Coconut flour offers various health‐promoting properties, including prebiotic, antihistamine, antibacterial, and anti‐COVID‐19 effects that contribute to a stronger immune system. Notably, this gluten‐free flour provides comparable nutritional value to wheat flour, lacking anti‐nutrients that can hinder mineral and protein absorption, such as phytic acid, lathyrogen, saponin, lecithin, hemagglutinin, and alpha‐amylase inhibitors. Its high fiber content makes it a valuable asset for promoting community health. As a result, it is a popular choice in gluten‐free bakeries, confectioneries, extruded products, snacks, and desserts. Furthermore, coconut flour production presents a sustainable solution, utilizing waste and byproducts from coconut milk and de‐oiling industries, which minimizes environmental impact. Additionally, coconut flour is cost‐effective to produce at any scale, and the production process requires basic and economical equipment (Ghorbannezhad, Derakhshan, and Daneshfard 2022; Kaur et al. 2019; Ramaswamy 2014; Rastogi 2019; Satheesh 2015; Trinidad et al. 2006).

Cakes are among the most beloved confections worldwide. The global cake market is projected to reach an impressive US$75 billion by 2023, with an estimated annual growth rate of 3.3% (Konstantas, Stamford, and Azapagic 2019). The type and quantity of ingredients used in a cake significantly influence its physicochemical and sensory properties, determining its texture, color, and overall quality (Conforti 2014). Studies have investigated the impact of adding coconut flour to different baked products. A study by Srivastava and Semwal (2015) suggested that substituting 5%–25% of wheat flour with coconut flour in cakes can produce a more viscous and denser batter, a redder and yellower crust, and a firmer texture. Also, muffins containing 20%–25% coconut flour achieved the highest score for overall consumer preference (Ramya and Anitha 2020). Studies on biscuits and cookies indicated increased consumer sensory acceptance and higher protein, fiber, and mineral content, along with a decrease in carbohydrates (particularly starch) when replacing up to 40% of wheat flour with coconut flour (Marikkar 2007; Sujirtha and Mahendran 2015). Studies by Stoin et al. (2020) and Paucean et al. (2016) explored the impact of substituting 45%–50% of rice flour with coconut flour in gluten‐free cookies, enhancing their physicochemical, nutritional, and sensory properties. Turabi, Sumnu, and Sahin (2008) investigated how the amount and combination of hydrocolloids and emulsifiers affect gluten‐free rice cakes. Their findings suggested that incorporating 1% xanthan gum significantly increased the cake's apparent viscosity, specific volume, firmness, and batter emulsion stability without affecting its specific gravity.

The Response Surface Method (RSM) is a highly effective approach for optimizing added and substituted formulations. The D‐optimal method maximizes the determinant of the intended matrix, thereby minimizing the total variance of the projected regression correlation coefficient. This approach's significant advantage is its ability to handle unconventional designs. Furthermore, RSM mixture design significantly reduces the number of experiments and replications required, leading to considerable time and resource savings (Mohamad Zen et al. 2015).

Our research aims to fill a crucial gap in gluten‐free baked products by developing a high‐quality cake tailored for individuals with CD, NCGS, and WA. While previous valuable studies have examined different gluten‐free formulations, mostly one‐variable‐at‐a‐time (OVAT), there has been limited focus on optimizing combinations of interdependent variables using regression modeling to predict dependent variables, particularly at high levels of RF, CF, and XG substitution ranges (50% RF/CF and 1% RF/XG ratio). To address this, we utilized a special cubic D‐optimal mixture design combined with advanced regression modeling, allowing us to investigate higher‐degree interactions beyond quadratic models. This method enabled us to systematically study the effects of these interdependent ingredients on the cake's physicochemical properties and sensory characteristics. By utilizing these cost‐effective and underutilized by‐products, our study not only enhances the cake's nutritional profile and reduces starch content (by RF substitution) but also provides a robust modeling framework for future gluten‐free cakes. Our research seeks to answer the key question: Can this experiment design (DOE) approach, combined with advanced modeling, predict an optimized ingredient substitution formula to produce a gluten‐free cake that meets dietary needs and aligns with global cake consumption trends? This work significantly contributes to the field by providing a scientifically validated method for creating superior gluten‐free products and advancing statistical modeling techniques in food science.

## 2 Materials and Method

### 2.1 Ingredients

The gluten‐free cake recipe utilized the following ingredients: Sadri Hashmi rice flour (RF) (local market, Iran) with a particle size of less than 420 μm, Extra fine‐grained desiccated low‐fat coconut flour (CF) (Setareh Nik Damavand importer Company, Indonesia) with a particle size of 710–850 μm, Sugar (Ofogh Doryan Samin, Iran), Isolated whey protein (DMV International, Netherlands), Vanilla essence (Mane, France), Xanthan gum (XG) (Fufeng, China), Water, Oil (Ladan Oil, Iran), Whole eggs (Telavang, Iran), Gel cake (emulsifiers blend), and Baking powder and inverted sugar (Azarnoosh Shokoufeh, Iran).

### 2.2 Methods

#### 2.2.1 Cakes Preparation

The ingredients were combined using the all‐in‐one method and mixed at high speed for 6 min in a 5‐L mixer bowl (Berjaya, Malaysia) to create the batter. Approximately 350 g of batter was poured into a greased pan and baked for 30 min at 180°C in a semi‐industrial oven (Convection, France). After cooling for 1 h, the 5 × 5 × 5 cm cakes were placed in low‐density polyethylene containers for further analysis. The physical properties of the batter were also measured after preparation.

#### 2.2.2 Rice and Coconut Flour Properties

The ash content (AACC 2000a), crude fat content (AACC 2000b), crude fiber content (AACC 2000c), and crude protein content (AACC 2000d) were determined using the nitrogen‐to‐protein factor of 5.75 for rice (Fujihara et al. 2008) and 5.3 for coconut flour (Sinaga, Margata, and Silalahi 2015), pH level (AACC 2000e), and moisture content (AACC 2000g) of rice and coconut flour were assessed by the guidelines provided by the American Association of Cereal Chemists (AACC).

#### 2.2.3 Batter Physical Properties

##### 2.2.3.1 Viscosity

Due to the batter's high viscosity, a Brookfield viscometer (Brookfield DV‐I Prime, US) equipped with the LV‐4 (64) spindle was used to measure its viscosity at 100 rpm. The results were recorded in centipoise (Ebrahimi, Tarzi, and Asghari 2016).

##### 2.2.3.2 Specific Weight

A gravimetric method was employed to determine the cake batter's specific weight. A 100‐mL measuring steel container was employed to establish a reference weight based on the weight of water measured at 25°C. The weight of the batter measured using the same container was then utilized in the following formula to calculate the batter's specific weight (AACC 2000h):

$$\text{Specific weight}=\frac{\text{batter weight}\;\left(g\right)}{\text{water weight}\;\left(g\right)}$$

#### 2.2.4 Cakes' Physicochemical Properties

##### 2.2.4.1 Colorimetry

The color of the cake crumbs was measured using a portable colorimeter (TES‐135, Taiwan) to explore the impact of various factors. Colorimetry was conducted on uniformly mixed cake crumb samples. Brightness (ΔL*), redness–greenness (Δa*), and yellowness–blueness (Δb*) were employed to compare and report color differences. The standard device's white (L* = 100) and black (a* = 0 and b* = 0) calibration plates were utilized to determine brightness and color variances. Color change differences were calculated using the following formula (Al‐Baarri et al. 2021; Srivastava and Semwal 2015).

$$\Delta E^{*}=\surd \left[{\left(\Delta L^{*}\right)}^{2}+{\left(\Delta a^{*}\right)}^{2}+{\left(\Delta b^{*}\right)}^{2}\right]$$

##### 2.2.4.2 Texture Profile Analysis (TPA)

TPA was operated with a texture analyzer (Brookfield CT V1.5, US) with a 10,000‐g load cell and TA5 standard probe to evaluate the cake's hardness. The compression target was set at 50% of the cake's 20 mm thickness, and the test speed was 1 mm/s (AACC 2000f; Wu et al. 2020). The first cycle of compression hardness (*N*) was recorded as the hardness response.

##### 2.2.4.3 Volume Index

The cake volume index was calculated using a standard ruler. The heights of the middle cut (*X*) and 2.5 cm from the center on both the left (*Y*) and right (*Z*) sides of the cake were measured. These measurements were used in the following equation to determine the volume index (AACC 2000i).

$$\text{Volume index}\;\left(\mathrm{cm}\right)=X+Y+Z$$

##### 2.2.4.4 Water Activity (*a*_*w*_)

Samples were placed in an *a*_*w*_ meter chamber (Novasina ms 1 set *a*_*w*_, Switzerland) at 25°C. Once the water vapor pressure stabilized, the sample's *a*_*w*_ at 25°C was determined using the following formula (Wu et al. 2020):

$$\text{Water activity}=\frac{\text{water vapor pressure of sample in}\;25{}^{\circ}C}{\text{pure water vapor pressure in}\;25{}^{\circ}C}$$

##### 2.2.4.5 Moisture Content

A 5‐g sample was weighed and then dried in an oven at 130°C ± 1°C for 60 min. After drying, the sample was transferred to a desiccator and held for 45–60 min until it reached room temperature. The moisture content was calculated using the following formula (AACC, 2000g):

$$\text{Moisture}\%=\frac{\text{water weight loss after drying}\;\left(g\right)}{\mathrm{wet}\;\text{sample}\;\left(g\right)}\times 100$$

#### 2.2.5 Sensory Evaluation

Ten trained panelists, aged 25–60, assessed the cake's color, taste, texture, and overall acceptance using a 9‐point hedonic scale (1 = lowest, 9 = highest) (Lim 2011). Each panelist evaluated 17 samples at room temperature and had access to water to cleanse their palates between tastings to prevent taste carryover. All panelists were informed about the ingredients before the evaluation and provided written consent. As the panelists were not a vulnerable population, formal ethics approval was not required for this evaluation. The average scores for each category were used for analysis.

### 2.3 Experimental Design

#### 2.3.1 D‐Optimal Mixture Design

The study assessed the direct and interactive effects of interdependent variables—RF (A), CF (B), and XA (C)—using a D‐optimal mixture design. After conducting preliminary formulation analysis and pre‐tests, it was determined that the minimum and maximum substitution levels for these variables were as follows: RF (15.60%–31.19%), CF (0%–15.59%), and XA (0%–0.31%) based on the batter weight formulation percentage (Table 1). To assess the interaction among all three variables simultaneously and their individual and pairwise effects, a special cubic Scheffé polynomial model for a D‐optimal mixture design was applied, with five replicates and five lack‐of‐fit points (Cornell 2002; Khuri and Mukhopadhyay 2010; Myers, Montgomery, and Anderson‐Cook 2016), resulting in a total of 17 experimental runs (Table 2). A comprehensive responses (dependent variables) analysis was conducted to address the research question of whether an optimized gluten‐free cake with the highest achievable quality properties and consumer acceptance could be developed within specified substitution ranges. This analysis evaluated the crucial batter's physical properties, the cake's physicochemical characteristics, and sensory attribute characteristics. Eleven responses were measured to ensure the final product meets both physicochemical and sensory quality standards, including batter viscosity, specific gravity, crumb color Δ*E*, *a*_*w*_, moisture content, texture hardness, volume index, and four sensory attributes (color, flavor, texture, and overall acceptability). Then, responses were regression modeled accordingly, allowing us to investigate the optimum combination of interdependent variables (A, B, C) to produce a high‐quality, consumer‐friendly gluten‐free cake.

**TABLE 1:** Gluten‐free rice‐based cake ingredients and formulation.

Ingredients	Batter weight %	Variables		
		Low %	High %	Substitution range %
A	A + B + C = 31.190	15.600	31.190	50
B		0	15.590	50
C		0	0.310	1
Sugar	10.397			
Whole egg	31.192			
Oil	8.318			
Water	13.516			
Baking powder	0.832			
Isolated whey protein	0.832			
Gel cake	1.497			
Inverted sugar	1.497			
Vanilla essence	0.728			
Total	100%			

*Note:* Variables are Sadri Hashemi rice flour (A), low‐fat desiccated coconut flour (B) and xanthan gum (C).

**TABLE 2:** Special cubic D‐optimal mixture design for interdependent variables.

Run order	Standard order	Actual component			L_Pseudo coded component		
		A	B	C	A	B	C
16	1	0.000	0.000	31.190	1.000	0.000	0.000
1	2	0.000	0.000	31.190	1.000	0.000	0.000
15	3	0.000	7.800	23.390	0.500	0.500	0.000
5	4	0.000	7.800	23.390	0.500	0.500	0.000
11	5	0.000	15.590	15.600	0.000	1.000	0.000
14	6	0.000	15.590	15.600	0.000	1.000	0.000
13	7	0.100	10.290	20.800	0.334	0.660	0.006
2	8	0.160	0.000	31.030	0.990	0.000	0.010
12	9	0.230	3.860	27.100	0.738	0.248	0.015
7	10	0.230	11.500	19.460	0.248	0.738	0.015
17	11	0.310	0.000	30.880	0.980	0.000	0.020
6	12	0.310	0.000	30.880	0.980	0.000	0.020
8	13	0.310	5.090	25.790	0.654	0.326	0.020
10	14	0.310	7.640	23.240	0.490	0.490	0.020
3	15	0.310	10.190	20.690	0.326	0.654	0.020
9	16	0.310	15.280	15.600	0.000	0.980	0.020
4	17	0.310	15.280	15.600	0.000	0.980	0.020

*Note:* Variables are Sadri Hashemi rice flour (A), low‐fat desiccated coconut flour (B), and xanthan gum (C). The 17 runs (formulations) of the matrix design are shown in terms of an actual component and L_Pseudo‐coded design.

#### 2.3.2 Model Fitting

The special cubic mixture model was selected to assess the combined substitution effects of the three interdependent variables (ABC) and potential third‐order effects that cannot be captured by linear or quadratic models, which are not designed for three‐way interactions. This model was utilized per Equation (1) (Cornell 2002; Myers, Montgomery, and Anderson‐Cook 2016). Furthermore, owing to its third‐degree nature, it also facilitated the assessment of other third‐degree terms to determine the best‐fit response variables in the proposed model.

(1) $$Y=b_{1}A+b_{2}B+b_{3}C+b_{12}\mathrm{AB}+b_{13}\mathrm{AC}+b_{23}\mathrm{BC}+b_{123}\mathrm{ABC}$$

where *Y* represents the response variable measured for each of the 17 formulations, the variables *b*_1_, *b*_2_, and *b*_3_ correspond to the regression coefficients for RF (A), CF (B), and XA (C), respectively. Furthermore, *b*_12_, *b*_13_, *b*_23_, and *b*_123_ denote the interaction coefficients for RF × CF (AB), RF × XA (AC), CF × XA (BC), and RF × CF × XA (ABC), respectively.

In order to evaluate the outcomes of each response and confirm their compatibility with the model, the following procedures were implemented: (I) The selection of the models was based on specific criteria, including a statistically significant model (*p* < 0.0001) with a non‐significant lack of fit (*p* > 0.1). Furthermore, preference was given to models with superior regression and fit quality, as evidenced by a higher coefficient of determination (*R* ^2^ > 0.85), higher adjusted *R* ^2^ (Adj‐*R* ^2^), and notably higher predicted *R* ^2^ (pred‐*R* ^2^) with a difference of less than 0.2 with Adj‐*R* ^2^, indicating models not overfitting (Montgomery 2020). Other selection criteria encompassed a higher adequate precision (> 4), indicating a favorable signal‐to‐noise ratio and a lower predicted residual sum of squares (PRESS) in comparison to other proposed models. These considerations were pivotal in defining the model's effectiveness in accommodating the proper response data fitting and errors. Models with marginally not‐significant lack of fit (0.05 < *p* < 0.1) were deemed unacceptable, as they did not inspire enough confidence in the model's fit. Following the Fisher's *F*‐test in the analysis of variance (ANOVA), non‐hierarchical terms that were confidently not significant (*p* > 0.1) were removed to optimize the models. Marginally significant terms (0.05 < *p* < 0.1) remained in the model due to the even minimal interaction effects, contributing to the fit and prediction capability of the regression models to explore subtle interaction effects and reduction of type II errors (Greenland et al. 2016; Morganstein and Wasserstein 2014). Additionally, hierarchical terms were retained in the model, regardless of their significance (*p* > 0.1), to maintain its degree and stability. Assumed parametric nature of all responses (average score from 10 evaluators for the four sensory analyses) necessitated validation to confirm normal distribution. This was achieved using a normal probability of residuals plot (should not be S shape), which was confirmed by conducting the Shapiro–Wilk normality test (*p* > 0.05). Additionally, for variance study (Homoscedasticity) and independence of errors, an external studentized residuals vs. predicted and vs. run plot was employed to ensure constant variance and independence of errors, respectively. Residuals were distributed within the range of +4 to −4 without any discernible pattern, thereby confirming the accuracy of the model's statistical tests and the data's variance and verifying the absence of systematic error (Encina‐Zelada et al. 2019; Vera Candioti et al. 2014). A Box‐Cox analysis was conducted to ensure better normality of distribution, which further improved the fitting for sensory response models. The analysis recommended utilizing a power and square root transformation function applied to the taste and overall acceptability models, respectively (Table 3).

**TABLE 3:** Responses models summary statistics of the analysis of variance (ANOVA), fit‐statistics, and equations in terms of the actual component.

	ANOVA *F*‐value (*p‐value*)											Fit‐statistic						
Dependent responses	Model type	Model	Linear mixture	AB	AC	BC	ABC	AB (A−B)	AC (A−C)	BC (B−C)	Lack of fit	CV%		*R* ^2^	Adj‐*R* ^2^	Pred‐*R* ^2^	AdPrec	PRESS
Viscosity (cP)	Special Cubic	170.06 (< 0.0001)	490.62 (< 0.0001)	5.94 (0.0350)	15.43 (0.0028)	15.08 (0.0030)	5.15 (0.0467)	—	—	—	0.6298 (0.6879)	4.12		0.9903	0.9845	0.9691	37.2991	2.592E+06
		*Y*_1_ = (90.4652 × A) + (471.332 × B) + (−968,883 × C) + (−9.45989 × A × B) + (31,893.2 × A × C) + (30,899.9 × B × C) + (41.1516 × A × B × C)																
Specific weight	Cubic	171.42 (< 0.0001)	703.43 (< 0.0001)	9.61 (0.0173)	8.41 (0.0230)	8.34 (0.0234)	8.31 (0.0236)	3.91 (0.0885)	8.43 (0.0229)	8.19 (0.0243)	3.02 (0.1382)	0.9773		0.9955	0.9897	0.9799	38.2677	0.0014
		*Y*_2_ = (0.0197152 × A) + (0.0475267 × B) + (−9564.41 × C) + (−0.00157106 × A × B) + (462.466 × A × C) + (460.452 × B × C) + (−9.92527 × A × B × C) + (2.72153e‐05 × A × B × (A−B)) + (−4.99613 × A × C × (A−C)) + (−4.9301 × B × C × (B−C))																
Δ*E*	Cubic	29.84 (< 0.0001)	106.27 (< 0.0001)	3.09 (0.1221)	5.62 (0.0496)	5.58 (0.0502)	5.55 (0.0506)	5.67 (0.0488)	5.61 (0.0498)	5.50 (0.0515)	0.2743 (0.7708)	0.8731		0.9746	0.9419	0.8743	18.4065	9.55
		*Y*_3_ = (1.90438 × A) + (3.36455 × B) + (605,614 × C) + (−0.105843 × A × B) + (−29267.6 × A × C) + (−29,188 × B × C) + (629.181 × A × B × C) + (0.00254099 × A × B × (A−B)) + (315.867 × A × C × (A−C)) + (313.242 × B × C × (B−C))																
*a*_*W*_	Reduced Cubic	72.26 (< 0.0001)	200.16 (< 0.0001)	0.0091 (0.9265)	23.69 (0.0012)	23.56 (0.0013)	23.64 (0.0013)	NS	23.84 (0.0012)	23.44 (0.0013)	0.3756 (0.7750)	0.0556		0.9863	0.9727	0.9311	25.0642	1.236E‐05
		*Y*_4_ = (0.0317884 × A) + (0.0319702 × B) + (1246.73 × C) + (7.42091e‐07 × A × B) + (−60.3148 × A × C) + (−60.1323 × B × C) + (1.29888 × A × B × C) + (0.652351 × A × C × (A−C)) + (0.646325 × B × C × (B−C))																
Moisture (%)	Special Cubic	70.15 (< 0.0001)	6.99 (0.0126)	210.91 (< 0.0001)	75.19 (< 0.0001)	75.58 (< 0.0001)	32.65 (0.0002)	—	—	—	2.75 (0.1453)	0.8499		0.9768	0.9629	0.9390	24.7750	2.28
		*Y*_5_ = (1.05768 × A) + (0.11381 × B) + (−2244.58 × C) + (0.0582717 × A × B) + (72.7936 × A × C) + (74.666 × B × C) + (−0.107163 × A × B × C)																
Hardness (*N*)	Reduced Cubic	147.02 (< 0.0001)	458.40 (< 0.0001)	106.68 (< 0.0001)	31.48 (0.0005)	31.42 (0.0005)	31.44 (0.0005)	NS	31.54 (0.0005)	31.35 (0.0005)	0.3785 (0.7731)	3.74		0.9932	0.9865	0.9608	33.9108	1.51
		*Y*_6_ = (0.0745915 × A) + (−0.2722 × B) + (−468,258 × C) + (0.0262645 × A × B) + (22642.5 × A × C) + (22615.1 × B × C) + (−488.375 × A × B × C) + (−244.633 × A × C × (A−C)) + (−243.723 × B × C × (B−C))																
Volume index (cm)	Linear	688.94 (< 0.0001)	688.94 (< 0.0001)	—	—	—	—	—	—	—	1.84 (0.2590)	0.8314		0.9899	0.9885	0.9855	73.2134	0.3117
		*Y*_7_ = (0.541024 × A) + (0.374835 × B) + (−3.59123 × C)																
Color acceptance	Reduced Cubic	392.51 (< 0.0001)	1399.75 (< 0.0001)	108.49 (< 0.0001)	43.77 (0.0002)	43.57 (0.0002)	43.53 (0.0002)	NS	43.83 (0.0002)	43.20 (0.0002)	2.48 (0.1763)		2.95	0.9975	0.9949	0.9498	61.3031	3.92
		*Y*_8_ = (0.24271 × A) + (−0.176915 × B) + (−482,395 × C) + (0.0231385 × A × B) + (23323.6 × A × C) + (23,263 × B × C) + (−501.973 × A × B × C) + (−251.958 × A × C × (A−C)) + (−249.96 × B × C × (B−C))																
(Taste acceptance)^2.46^	Cubic	1006.88 (< 0.0001)	3309.33 (< 0.0001)	1614.51 (< 0.0001)	203.88 (< 0.0001)	203.23 (< 0.0001)	204.64 (< 0.0001)	11.04 (0.0127)	205.54 (< 0.0001)	203.50 (< 0.0001)	0.2193 (0.8104)	4.04		0.9992	0.9982	0.9935	94.2908	445.18
		*Y*_9_ = (4.62911 × A) + (−38.1966 × B) + (−1.91539e+07 × C) + (2.43664 × A × B) + (926,775 × A × C) + (925,809 × B × C) + (−20,024.6 × A × B × C) + (−0.0185881 × A × B × (A−B)) + (−10026.8 × A × C × (A−C)) + (−9991.59 × B × C × (B−C))																
Texture acceptance	Reduced Cubic	1241.58 (< 0.0001)	4329.90 (< 0.0001)	457.50 (< 0.0001)	146.33 (< 0.0001)	147.19 (< 0.0001)	145.69 (< 0.0001)	NS	144.35 (< 0.0001)	146.94 (< 0.0001)	0.3279 (0.8062)	2.01		0.9992	0.9984	0.9964	101.8340	0.3468
		*Y*_10_ = (0.200295 × A) + (−0.199064 × B) + (−550,606 × C) + (0.02972 × A × B) + (26,572.8 × A × C) + (26,646.3 × B × C) + (−574.435 × A × B × C) + (−285.992 × A × C × (A−C)) + (−288.335 × B × C × (B−C))																
Sqrt (Overall acceptance)	Reduced Cubic	1408.82 (< 0.0001)	4386.23 (< 0.0001)	477.77 (< 0.0001)	30.15 (0.0006)	30.57 (0.0006)	29.86 (0.0006)	NS	29.21 (0.0006)	30.48 (0.0006)	0.6747 (0.6037)	1.04		0.9993	0.9986	0.9964	111.8858	0.0197
		*Y*_11_ = (0.0878304 × A) + (−0.0436977 × B) + (−55559.9 × C) + (0.0067673 × A × B) + (2675.43 × A × C) + (2694.09 × B × C) + (−57.9478 × A × B × C) + (−28.6677 × A × C × (A−C)) + (−29.2603 × B × C × (B−C))																

*Note:* The analysis of variance (ANOVA) presents *F*‐values and *p*‐values (in parenthesis) for the models, linear mixture terms, interaction terms, and lack‐of‐fit related to the physicochemical and sensory characteristics of batter and cake. Fit‐statistics, including Coefficient of Variation (CV%), Coefficient of Determination (*R* ^2^), Adjusted *R* ^2^ (Adj‐*R* ^2^), Predicted *R* ^2^ (Pred‐*R* ^2^), Adequate Precision (AdPrec), and Predicted Residual Error Sum of Squares (PRESS), are provided. The term “NS” denotes that the terms were not hierarchical and not significant (*p* > 0.1) and therefore eliminated. “—” indicates that interactions were not included in the model and not calculated. “Sqrt” denotes the square root. The interdependent variables are Sadri Hashemi rice flour (A), Low‐fat desiccated coconut flour (B), and xanthan gum (C).

#### 2.3.3 Optimization

**FIGURE 1:**
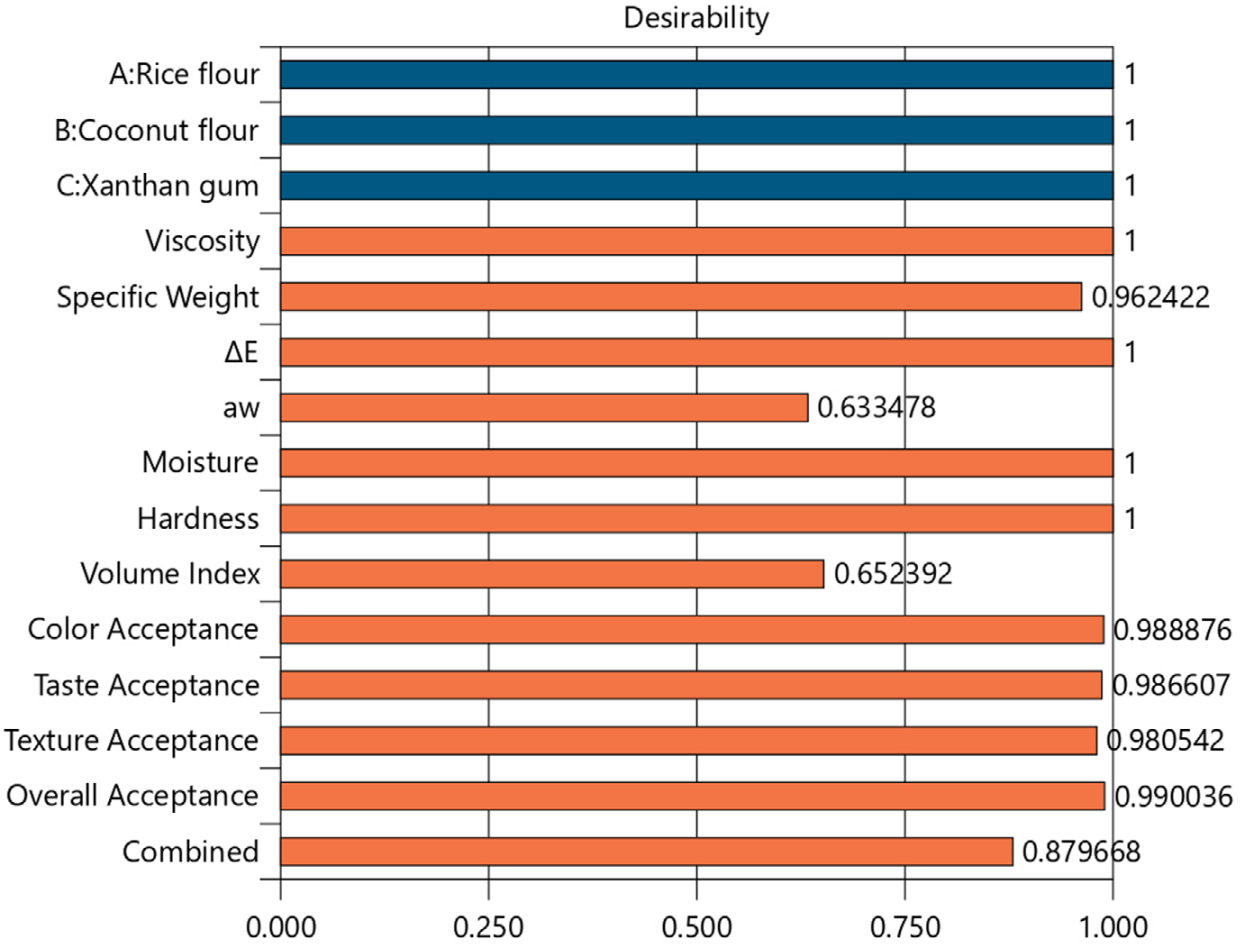
Individual and combined desirability score bar graph of numerical optimization.

A numerical desirability function was conducted after a thorough analysis and identification of the model for each response. This particular process determined the best combination of the three interdependent variables based on the qualitative and sensory properties of the cake. The desired constraint for each of the 11 responses was specified, and each response (*Y*) was transformed into a desirability function (*d*) ranging from 0 to 1, based on whether the goal was to minimize, in range, or maximize the response (Table 4). For responses to be maximized, a desirability value of 1 was assigned to higher and 0 to lower. Conversely, for minimization, the reverse was applied. Also, individual desirabilities were given the lower and upper weight and importance score (Table 4). Our objective was to develop the best possible recipe for a consumer‐friendly and widely favored cake, taking into account both its physical properties and sensory appeal. To do this, we operated to minimize the batter's specific weight and cake's *a*_*w*_, maximize the volume index and the four sensory acceptability factors, and ensure that the viscosity, moisture content, texture hardness, and crumb color Δ*E* all fell within the range. We also analyzed the viscosity, moisture content, texture hardness, and crumb color Δ*E* to assess the significant impact of substituting CF and XA and to identify any specific regression patterns. Additionally, we sought to determine their predictive accuracy for future studies by evaluating their pred‐*R* ^2^ values. The desirability function operates as an algorithm to determine the optimal range for each response point. Based on Derringer and Suich (1980) method, the final composite desirability index (Figure 1) is expressed in Equation (2) (Costa, Lourenço, and Pereira 2011; Encina‐Zelada et al. 2019).

(2) $$D=\left(\prod_{i=1}^{n}{d_{i}}^{\frac{I_{i}.w_{i}}{\sum_{i=1}^{n}I_{i}.w_{i}}}\right)=\text{or}\;\left(d_{1}^{\frac{I_{1}.w_{1}}{\sum I_{i}.w_{i}}}\times \;d_{2}^{\frac{I_{2}.w_{2}}{\sum I_{i}.w_{i}}}\times \ldots \times d_{n}^{\frac{I_{n}.w_{n}}{\sum I_{i}.w_{i}}}\right)$$

where *D* is expressed as the geometric mean of the composite desirability functions that correspond to response 1 (*d*_1_), response 2 (*d*_2_), response “*n*” (*d*_n_), and “*n*” is the total number of analyzed responses. The *d*_*i*_ represented the individual desirability for the *i*_th_ response, *I*_*i*_ indicated the importance of the *i*_th_ response, *w*_*i*_ represented weight for the *i*_th_ response, and ∑*I*_*i*_·*w*_*i*_ indicated the sum of the importance times weight for all responses. The algorithm should seek response variable values for which *D* approaches 1 (Costa, Lourenço, and Pereira 2011; Encina‐Zelada et al. 2019).

**TABLE 4:** Individual desirability function constraints and goals of optimization.

Dependent response	Goal	Lower limit	Upper limit	Lower weight	Upper weight	Importance
A: Rice flour	In range	15.6	31.19	1	1	3
B: Coconut flour	In range	0	15.59	1	1	3
C: Xanthan gum	In range	0	0.31	1	1	3
Viscosity (cP)	In range	2861	9889	1	1	3
Specific weight	Minimize	0.615	0.818	1	1	3
Crumb Δ*E*	In range	56.0589	63.7623	1	1	3
*a*_*W*_	Minimize	0.989	1	1	1	3
Moisture (%)	In range	32.374	36.736	1	1	3
Texture hardness (N)	In range	2.31	6.77	1	1	3
Volume Index (cm)	Maximize	13.1	16.9	1	1	3
Color acceptance	Maximize	1.3	8.6	1	1	3
Taste acceptance	Maximize	1.3	8.7	1	1	3
Texture acceptance	Maximize	1.2	8.7	1	1	3
Overall acceptance	Maximize	1.3	8.8	1	1	5

*Note:* The goals and importance score were chosen based on the cake's physicochemical properties and desired sensorial attributes. Lower and upper limits demonstrate that each response measured low and high data, and lower and upper weights indicate the given weight to each response's desirability function.

#### 2.3.4 Confirmation

Upon identifying the optimal sample with a desirability index exceeding 0.8 (80%), a two‐sided confirmation run test was conducted to validate the model's responses within the predicted interval (PI) at both upper and lower bounds with a 95% confidence level (*p* ≤ 0.05). Subsequently, the optimal sample was created, and all 11 tests (responses) were repeated, with three replicates for the qualitative tests and 10 replicates (involving 10 panelists) for the sensory evaluations. The mean results obtained from the three and 10 replicates, respectively, were required to fall within the predicted interval (PI) range—both upper and lower limits—at a 95% confidence level to confirm the accuracy and alignment of the model with the actual outcomes (Table 5). This meticulous process ensures that the model predictions are not only statistically robust but also practically reliable in real‐world applications, thereby further validating the accuracy of the response predictions. Finally, the mixture design, ANOVA, fit‐statistic, diagnostics plot, box‐cox transformation, optimization, confirmation, and two component effects and 3D surface graphs were conducted using the free trial Design Expert version 13.0.5.0 (Stat‐Ease, USA).

**TABLE 5:** Models validation using a two‐sided confirmation run with 95% confidence (*p* ≤ 0.05) between the high and low predicted intervals (PI).

Dependent responses	Predicted mean	Std.dev	*n*	SE Pred	95% PI low	Data mean	95% PI high
Viscosity (cP)	4065.320	285.193	3	253.833	3499.750	4235.000	4630.900
Specific weight	0.623	0.007	3	0.006	0.608	0.620	0.637
Crumb Δ*E*	58.706	0.525	3	0.478	57.576	59.559	59.837
*a*_*W*_	0.993	0.001	3	0.001	0.992	0.992	0.994
Moisture (%)	36.258	0.295	3	0.262	35.673	36.025	36.843
Texture Hardness (N)	4.410	0.180	3	0.160	4.040	4.170	4.790
Volume Index (cm)	15.580	0.120	3	0.090	15.390	15.550	15.760
Color acceptance	8.5	0.2	10	0.1	8.2	8.3	8.8
Taste acceptance^a^	8.7	0.0	10	N/A^b^	8.6	8.6	8.7
Texture acceptance	8.6	0.1	10	0.1	8.4	8.5	8.7
Overall acceptance^a^	8.7	0.1	10	N/A^b^	8.5	8.7	8.9

*Note:* The data means all fell within the upper and lower predicted intervals, indicating that the models developed for the physicochemical and sensory properties of the batter and cake have been validated. This confirms that the models have good predictive capability. The mean data is expressed as mean (*n* = 3 and 10). Standard deviation (Std. dev) and predicted standard error (SE pred) represent the variability and precision of the validated response models, respectively.

^a^ Indicates the data mean is calculated on the transformed scale for transformed responses.

^b^ Denotes that the standard error (SE) could not be predicted on the original scale.

## 3 Results and Discussion

Tables 6 and 3 have demonstrated RF and CF physicochemical properties and responses models ANOVA, fit‐statistics, and models' equations regarding actual components, respectively.

**TABLE 6:** Physicochemical properties of Sadri Hashemi rice flour and low‐fat desiccated coconut flour.

	Sadri Hashemi rice flour (Mean ± Std.dev)	Low‐fat desiccated coconut flour (Mean ± Std.dev)
Ash %	0.336 ± 0.024	0.975 ± 0.091
Moisture %	6.735 ± 0.409	1.018 ± 0.056
pH	6.224 ± 0.033	6.086 ± 0.076
Crude protein %	7.570 ± 0.322	5.741 ± 0.113
Crude fat %	1.381 ± 0.192	55.971 ± 0.549
Crude fiber %	5.864 ± 0.710	39.103 ± 1.925

*Note:* Data are expressed as mean ± Standard deviation (*n* = 3).

### 3.1 Batter's Physical Properties

#### 3.1.1 Viscosity

**FIGURE 2:**
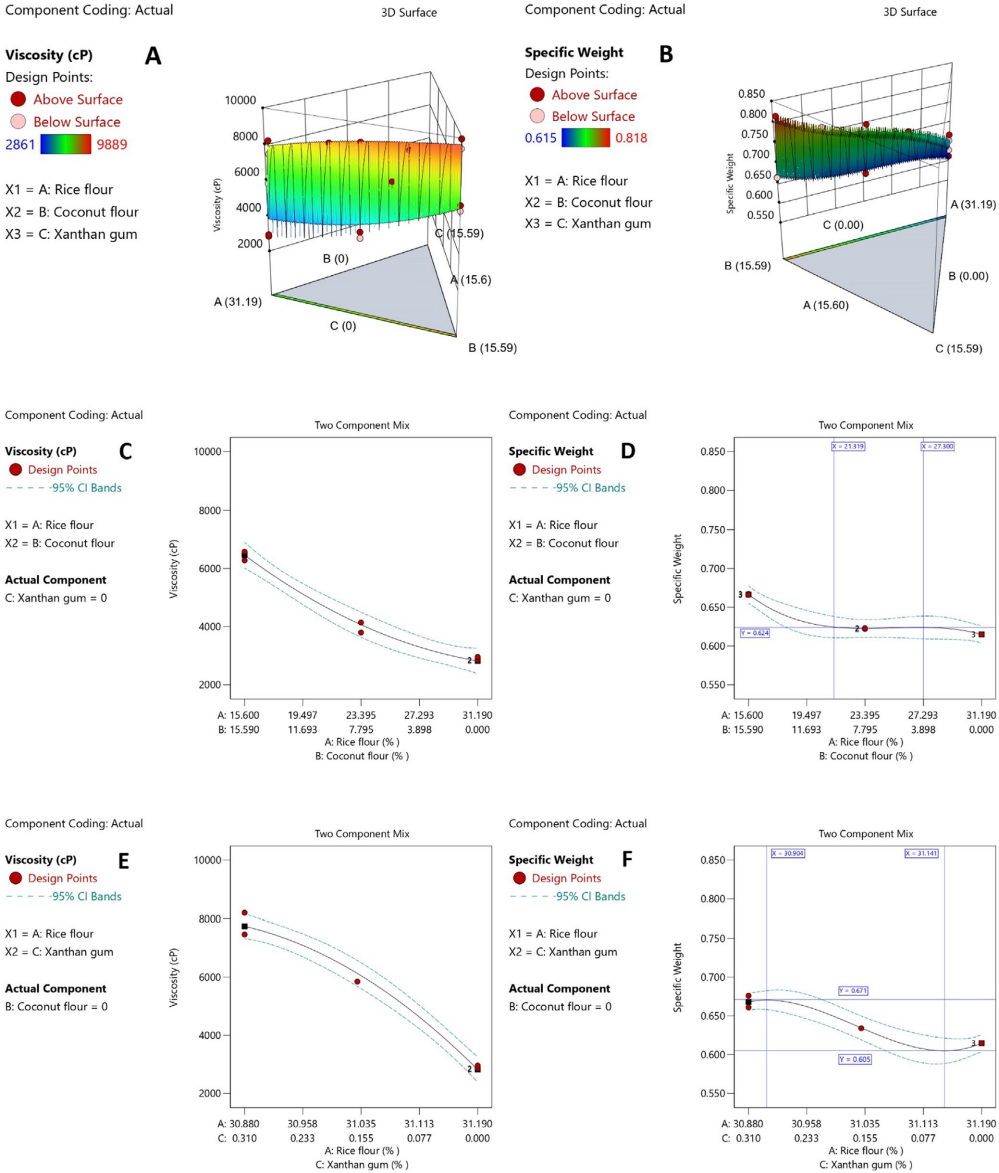
3D contour surface (3 variables interaction) and two component effects on batter physical properties. (A) Viscosity 3D surface; (B) Specific weight 3D surface; (C) RF‐CF effect on viscosity when XG constantly 0%; (D) RF‐CF effect on specific weight when XG constantly 0%; (E) RF‐XG effect on viscosity when CF constantly 0%, and (F) RF‐XG effect on specific weight when CF constantly 0%. RF (Rice Flour), CF (Coconut Flour), and XG (Xanthan Gum).

The RF substitution with CF and XG increased batter viscosity (*p*_ABC_ = 0.0467), as illustrated in Figure 2A. Maximum CF and XG substitution led to the highest viscosity and vice versa. Our findings (*p*_AB_ = 0.0350) were consistent with those of Aydogdu, Sumnu, and Sahin (2018) and Srivastava and Semwal (2015), who observed a significant viscosity increase upon adding fibers. Similarly, Turabi, Sumnu, and Sahin (2008) demonstrated a significant viscosity increase with a 1% XG addition to rice cakes, supporting our results (*p*_AC_ = 0.0028). The combination of water‐absorbing CF, a suitable substitute for low‐protein gluten‐free flours like RF, and thickening agent XG likely contributed to the exponential increase in batter viscosity observed in the special cubic model (Table 3), suggesting a synergistic effect (*p*_BC_ = 0.0030). Replacing either CF or XG while holding the other constant at 0% increased viscosity (Figure 2C,E). Batter viscosity and consistency directly impact the final product, particularly the cake volume. Less viscous batters lose air bubbles rapidly, leading to reduced cake volume. Conversely, excessively viscous batters hinder proper air bubble formation and distribution, reducing cake expansion. Optimal viscosity, therefore, allows the batter to retain more air bubbles, stabilizing the cake. While CF thickens the batter, its coarse particles prevent air bubble stability, resulting in a thicker batter without improved air retention (Aydogdu, Sumnu, and Sahin 2018; Gularte et al. 2012; Masoodi, Sharma, and Chauhan 2002; Srivastava and Semwal 2015). Conversely, adding XG to rice‐based gluten‐free cakes can improve stability and volume and create a porous texture (Encina‐Zelada et al. 2018; Preichardt et al. 2011; Sumnu et al. 2009; Turabi, Sumnu, and Sahin 2008).

#### 3.1.2 Specific Weight

The batter's specific weight directly influences the final cake's texture, volume, and quality. Factors such as ingredients, their quantities, and the mixing techniques used all contribute to the batter's specific weight, which is determined by air bubbles forming and cell sizes enlarging during mixing. More entrapped air bubbles result in a lower specific weight, making the cake lighter and softer. Conversely, a higher specific weight makes the cake denser and heavier (Ahmadi, Aghajani, and Gohari Ardabili 2022; Srivastava and Semwal 2015). Figure 2B showed that the batter's specific weight increased significantly (*p*_ABC_ = 0.0236) when CF and XG were simultaneously substituted for RF. Also, Figure 2D showed a slight increase in specific weight when the RF/CF ratio was increased from 0% to 3.898% without adding XG (0%). Within the 3.898%–7.795% CF substitution range, the specific weight initially increased slightly, then decreased slightly before rising significantly (*p*_AB_ = 0.0173) beyond 7.795%. An initial slight decrease could be attributed to adjustments in batter viscosity, which allowed proper air bubble retention. However, the coarse CF particles hindered air bubble retention, significantly increasing specific weight beyond the 7.795% replacement level. Unlike other flours or soluble fibers, CF lacks surface‐active agents and emulsifiers, preventing the formation of a stabilizing external lipid layer around air bubbles (Srivastava and Semwal 2015). Furthermore, the fiber's types and granular size could affect the batter's specific weight. Smaller fiber particles have less water‐holding capacity, impacting the batter's specific weight (Aydogdu, Sumnu, and Sahin 2018). Additionally, substituting RF with water‐absorbing CF, which simultaneously reduced starch content, increased the interfering interactions between CF's fiber and the starch/protein matrix, which heightened competition for water absorption, reducing starch swelling and air bubbles entrapment, ultimately increasing the specific weight (Gularte et al. 2012; Kırbaş, Kumcuoglu, and Tavman 2019; Ozyigit et al. 2020). Notably, when the CF's content was constant at 0%, substituting RF with XG (0%–0.049%) reduced the batter's specific weight. However, beyond 0.049%, the specific weight increased significantly (*p*_AC_ = 0.0230) (Figure 2F). As previously mentioned, adjustments in batter's viscosity and forming a starch/protein/XG matrix contributed to enhanced air bubble size and retention, resulting in a lower specific weight, consistent with Turabi, Sumnu, and Sahin's (2008) findings. In contrast to Turabi, Sumnu, and Sahin (2008), our findings indicated that specific weight increased beyond 0.049% XG substitution, possibly due to damaged rice kernels' starch granules, leading to reduced starch swelling and starch/protein matrix development and increased competition between high water‐absorbing XG and damaged starch granules for water absorption, as observed in the studies by Andrade et al. (2018) and Herranz et al. (2016). Ultimately, substituting CF and XG with RF resulted in opposing interactions between variables at certain points (*p*_BC_ = 0.0234), resulting in a full cubic model (Table 3).

### 3.2 Cake Physicochemical Properties

#### 3.2.1 Crumb Color

**FIGURE 3:**
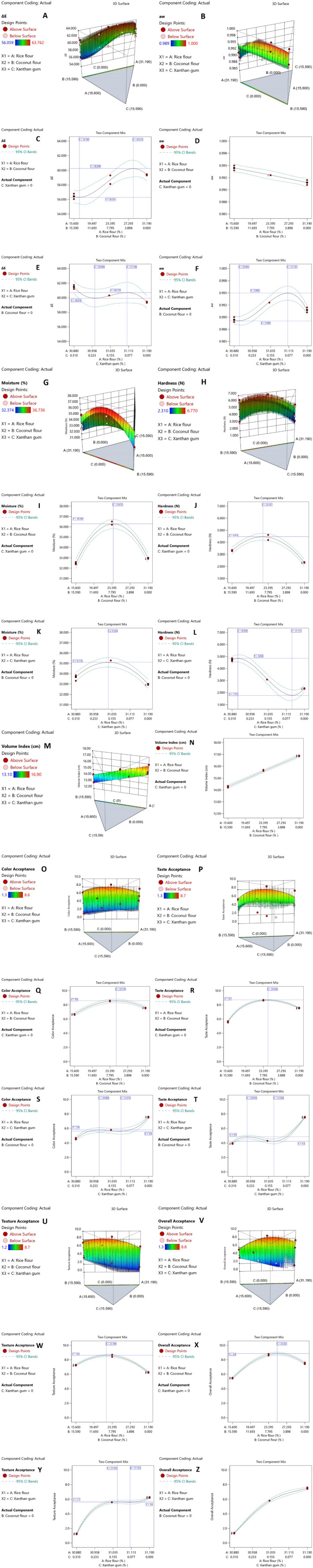
3D contour surface (3 variables interaction) and two‐component effects on cake physicochemical and sensory characteristics. (A) Δ*E* 3D surface; (B) *a*_*w*_ 3D surface; (C) RF‐CF effect on Δ*E* when XG constantly 0%; (D) RF‐CF effect on *a*_*w*_ when XG constantly 0%; (E) RF‐XG effect on Δ*E* when CF constantly 0%; (F) RF‐XG effect on *a*_*w*_ when CF constantly 0%; (G) Moisture 3D surface; (H) Hardness 3D surface; (I) RF‐CF effect on moisture when XG constantly 0%; (J) RF‐CF effect on hardness when XG constantly 0%; (K) RF‐XG effect on moisture when CF constantly 0%; (L) RF‐XG effect on hardness when CF constantly 0%; (M) Volume index 3D surface; (N) RF‐CF effect on volume index when XG is constantly 0%; (O) Color acceptance 3D surface; (P) Taste acceptance 3D surface; (Q) RF‐CF effect on color acceptance when XG constantly 0%; (R) RF‐CF effect on taste acceptance when XG constantly 0%; (S) RF‐XG effect on color acceptance when CF constantly 0%; (T) RF‐XG effect on taste acceptance when CF constantly 0%; (U) Texture acceptance 3D surface; (V) Overall acceptance 3D surface; (W) RF‐CF effect on texture acceptance when XG constantly 0%; (X) RF‐CF effect on overall acceptance when XG constantly 0%; (Y) RF‐XG effect on texture acceptance when CF constantly 0%; (Z) RF‐XG effect on overall acceptance when CF constantly 0%. RF (Rice Flour), CF (Coconut Flour), and XG (Xanthan Gum).

**FIGURE 4:**
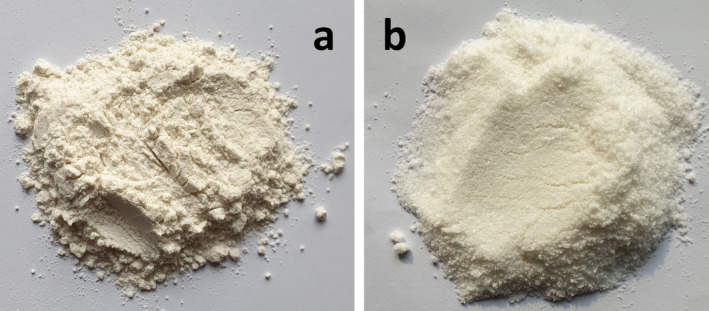
Sadri Hashemi rice flour (a) and low‐fat extra fine‐grained coconut flour (b) as flour ingredients.

Water, pH, sugar, amino acids, ingredients, baking time, and temperature affect the cake's final color. Mainly, Maillard and caramelization phenomena cause cake crust color variations. However, the color of the cake crumb, which was the focus of this study, is mainly determined by the inherent color of the ingredients and internal physicochemical reactions (Aydogdu, Sumnu, and Sahin 2018; Srivastava and Semwal 2015). Figure 3A has shown that substituting RF‐CF (5.090%) and RF‐XG (0.310%) increased Δ*E* significantly (*p*_ABC_ = 0.0506). However, Δ*E* remarkably decreased beyond these values. In detail, Figure 3C,E demonstrated substituting RF with CF about 0%–2.906% (XG 0%) and increasing XG 0%–0.084% (CF 0%), respectively, increased Δ*E* (darker (ΔL* ↑), more redness (Δa* ↑) and yellowness (Δb* ↑)), which is agreed with those Aydogdu, Sumnu, and Sahin (2018) who utilized 5% and 10% oat, pea, lemon, and apple fiber replaced for wheat flour, Singh, Liu, and Vaughn (2012) who substituted 0%–30% wheat flour with corn bran, Srivastava and Semwal (2015) who added 0%–20% coconut to wheat flour, and Gadallah et al. (2016) who utilized 2%, 3%, and 4% of XG added to 40% rice, 40% corn, and 20% potato starches. Increasing Δ*E* may be attributed to improved water dispersion facilitated by fibers and hydrocolloids in the cake, resulting in better thermal conduction. In contrast with the researchers above, Δ*E* decreased when over 2.906% CF and 0.084%–0.221% XG were incorporated. Furthermore, Δ*E* significantly (*p*_AC_ = 0.0496) increased in the 0.221%–0.31% XG range, resulting in a full cubic model with synergistic (*p*_BC_ = 0.0502) interactions. Variation in Δ*E* resulted from RF (darker (ΔL* ↑), greater redness (Δa* ↑) and yellowness (Δb* ↑)) and CF (brighter (ΔL* ↓), less redness (Δa*↓), and yellowness (Δb* ↓)) intrinsic colors (Figure 4), measured by white (L* = 100) and black (a* = 0 and b* = 0) standard sheets. Ultimately, CF's inherent color had more effects on crumb color change.

#### 3.2.2 Water Activity ( *a*_*W*_ )

The *a*_*w*_ of cake impacts its microbiological growth and storage. It is influenced by the cake's ingredients' water absorption and holding capacity, which are correlated to the moisture content (Alpers et al. 2021; Morassi et al. 2022; Noorlaila et al. 2017). Figure 3D illustrated that when RF was substituted with CF (0% XG), there was no significant water‐holding capacity (*p*_AB_ = 0.9265), which is supported by Singh, Liu, and Vaughn's (2012) research that included 0%–30% corn bran fiber in the cake. According to Aydogdu, Sumnu, and Sahin (2018), Singh, Kaur, and Singh (2016), and Srivastava and Semwal (2015), fibers, especially soluble fibers, enhance water absorption. However, in our study, finer CF particle size (Singh, Liu, and Vaughn 2012), CF high‐fat content based on Table 6 (lipophilic effect), CF high insoluble fiber (Adeloye, Osho, and Idris 2020), and the decrease in RF starch (which has an 83.2% water absorbing capacity) when substituted with CF (Remya and Jyothi 2015), reduced water holding capacity, increasing *a*_*w*_. Furthermore, XG increased the *a*_*w*_ in the range of 0%–0.083% due to increased water absorption (Figure 3F), as investigated by Hojjatoleslami and Azizi (2015) and Noorlaila et al. (2017). However, when the XG content exceeded 0.083%, the *a*_*w*_ decreased (*p*_AC_ = 0.0012) due to its strong water‐binding and holding capacity, supporting Encina‐Zelada et al.'s (2018) findings. Figure 3B showed that XG and CF may have competed for water retention at some points, exhibiting an antagonistic effect (*p*_BC_ = 0.0013). As the CF content increased, the ability of XG to retain water significantly decreased (*p*_ABC_ = 0.0013), which might be due to the reduced free water availability, resulting in a reduced cubic model (Table 3).

#### 3.2.3 Moisture Content

Moisture content plays a crucial role in cake consumers' acceptance. The evaporation of water during baking is influenced by the ingredients' water absorption and retention capacities, as well as the initial moisture content of the batter. As illustrated in Figure 3I, the moisture content increased significantly (*p*_AB_ < 0.0001) up to 7.518% RF‐CF ratio (0% XG) due to the high water‐absorption capacity of the coconut flour's fiber, which was consistent with the observations of Adeloye, Osho, and Idris (2020), Aydogdu, Sumnu, and Sahin (2018), Dhankhar and Tech (2013), Hossain et al. (2016), Mihiranie, Jayasundera, and Perera (2017), Srivastava and Semwal (2015), Sujirtha and Mahendran (2015), and Sundaresan et al. (2023). Conversely, the moisture content decreased significantly (*p*_AB_ < 0.0001) beyond 7.518% RF substitution with CF, which could be attributed to the lower CF moisture content compared to RF (Table 6), CF's finer particle size, CF's low soluble fiber (Adeloye, Osho, and Idris 2020; Dhingra et al. 2012; Singh, Liu, and Vaughn 2012), and RF's starch content reduction as substituted (Conforti 2014; Wilderjans et al. 2010), which led to reduced water retention, decreasing moisture. Additionally, substituting RF with CF likely reduced the water binding capacity due to a decrease in rice's hydrophilic water‐soluble albumin protein (Table 6 shows a higher nitrogen content in RF), resulting in greater moisture loss (Amagliani et al. 2017). The decrease in moisture content beyond the 7.518% RF substitution with CF was aligned with the findings of Singh, Liu, and Vaughn (2012) and Srivastava and Semwal (2015) on cake moisture loss increasing, bread water absorption decreasing (Gunathilake, Yalegama, and Kumara 2009), and cake and biscuit moisture content decreasing (Afoakwah, Owusu, and Owusu 2019; Makinde and Taibat 2018). Figure 3K has demonstrated that the substitution of 0%–0.170% RF with water‐absorbing hydrophilic XG (CF 0%) increased (*p*_AC_ < 0.0001) moisture as expected, supported by the findings of Gadallah et al. (2016) and Herranz et al. (2016). However, beyond a 0.170% replacement, less water might have been available for starch to absorb, resulting in the moisture content decreasing (Andrade et al. 2018). As shown in Figure 3G (*p*_ABC_ < 0.0002), increasing XG by 0.152% had the most synergistic effect (*p*_BC_ < 0.0001) on moisture content at every RF‐CF substitution point. However, exceeding 0.152% XG resulted in an antagonistic influence on moisture content, leading to a special cubic model (Table 3).

#### 3.2.4 Texture Hardness

The cake's hardness is influenced by the composition of the ingredients and the effectiveness of air bubble retention and distribution during mixing and baking. Generally, hardness is inversely linked to the cake's specific volume, which, in turn, is directly related to the viscosity and specific weight of the batter (Aydogdu, Sumnu, and Sahin 2018; Srivastava and Semwal 2015). The regression analysis in Figure 3J indicated that substituting RF with CF up to 8.944% led to a significant increase in hardness (*p*_AB_ < 0.0001), which could be attributed to coarse particles and the absence of surface‐active agents in CF, which hindered gas retention by increasing the batter's viscosity and specific weight (Srivastava and Semwal 2015). Hardness‐increasing results were aligned with the findings of Aydogdu, Sumnu, and Sahin (2018), Gularte et al. (2012), Sharoba, Farrag, and Abd El‐Salam (2013), Singh, Liu, and Vaughn (2012), and Srivastava and Semwal (2015), all of whom incorporated different fiber sources into the baked and confectionery products. CF's high water absorption and low holding capacity likely reduced available water for starch and soluble proteins to absorb, resulting in increased moisture loss and hardness after baking. Conversely, substituting more than 8.944% RF with CF reduced hardness, possibly due to the high‐fat content of CF (Table 6), which lubricated the cake structure (Dhankhar and Tech 2013). Additionally, more than 8.944% RF substitution with CF could have decreased hardness by starch content reduction and more water absorption. Similar results were reported by Dhankhar and Tech (2013), Srivastava (2010), and Sujirtha and Mahendran (2015), who observed that adding CF to wheat flour decreased cookie and biscuit hardness. Notably, we observed that a large CF substitution resulted in an inconsistent and sandy texture, potentially due to insufficient water for starch granules to swell and gelatinize properly, resulting in a poor texture. Figure 3L has demonstrated that when CF content remained constant at 0%, increasing the amount of XG from 0% to 0.053% significantly decreased hardness (*p*_AC_ = 0.0005), which could be attributed to XG's ability to adjust viscosity, facilitating air entrapment, and reducing the batter's specific weight. Moreover, the appropriate use of hydrocolloids could postpone starch retrogradation by retaining moisture and weakening the starch network, which occurs by preventing the formation of intraparticle junctions within the amylose chain, ultimately enhancing water distribution and reducing hardness (Mohammadi et al. 2014). In contrast, substituting 0.053%–0.285% RF with XG increased hardness, possibly due to reduced air and water retention, which might be attributed to reduced starch swelling and amylose leaching from granules, potentially strengthening and stabilizing the starch‐protein‐XG network through ionic interactions between XG's carboxylate groups and strong polymeric interactions (Mohammadi et al. 2014; Noorlaila et al. 2017). Furthermore, the augmented thickness of the crumb wall, which encases the gas cells in the cake, may have contributed to the increased hardness (Turabi, Sumnu, and Sahin 2008). These findings were consistent with previous studies by Andrade et al. (2018), Encina‐Zelada et al. (2018), Gularte et al. (2012), Mezaize et al. (2009), Noorlaila et al. (2017), and Turabi, Sumnu, and Sahin (2008), in which they indicated hardness increasing in XG‐utilized wheat‐based cakes, gluten‐free cakes, and French‐style bread. Finally, the concurrent substitution of RF with both CF and XG led to a synergistic increase in hardness (*p*_BC_ = 0.0005) at each interaction point, resulting in a reduced cubic model (*p*_ABC_ = 0.0005, Figure 3H).

#### 3.2.5 Volume Index

The presence and retention of gas bubbles during the mixing and baking processes significantly impact the cake's volume index, closely linked to the batter's viscosity and specific weight and the raw ingredients' inherent properties, playing a crucial role in determining the final product's quality. According to the linear prediction presented in Figure 3N, a significant decrease (*p*_B_ < 0.0001) in the volume index was observed with an increase in the proportion of RF substituted with CF, which can be attributed to the presence of coarse particles and the absence of surface‐active agents in CF (Srivastava and Semwal 2015), hindering the stabilization of air bubbles, ultimately leading to a reduction in the volume index. Furthermore, CF was found to interfere with the starch‐protein matrix, weakening the network responsible for retaining air bubbles and causing a decrease in the volume index (Aydogdu, Sumnu, and Sahin 2018). Our findings were supported by the observations of Masoodi, Sharma, and Chauhan (2002) and Srivastava and Semwal (2015), who reported a reduction in the volume index of wheat‐based cakes when using apple pomace and CF, respectively. An increase in RF substitution with XG also decreased the volume index (*p*_c_ < 0.0001), which could be attributed to XG's ability to restrict the expansion of air bubbles during baking. These results were consistent with the findings of Andrade et al. (2018), Lazaridou and Biliaderis (2009), Mezaize et al. (2009), and Xian and Hu (2018), who observed a decrease in specific volume (related to volume index) in cakes and French‐style bread while increasing XG content. However, Turabi, Sumnu, and Sahin (2008) reported an increase in the volume index for rice‐based gluten‐free cakes with 1% XG incorporation. The resultant contradiction may stem from various factors, such as differences in rice types, the amylose and amylopectin amount in the rice flour utilized, the type and quantity of baking powder employed, baking duration and temperature, damaged starch granules, and the emulsifiers applied. In Figure 3M, a linear regression prediction indicated a significant decrease in the volume index (*p*_volume index_ < 0.0001) when substituting RF with CF and XG.

#### 3.2.6 Sensory Evaluation

In Figure 3Q, it was observed that the color sensory evaluation of the cake (*p*_AB_ < 0.0001) aligned with the findings of Singh, Liu, and Vaughn (2012), Srivastava and Semwal (2015), and Hossain et al. (2016) when the RF substitution with CF was in the range of 0%–6.415% (0% XG). Additionally, our study confirmed the observations of Andrade et al. (2018), indicating that as XG increased at a constant 0% CF (Figure 3S), the color sensory acceptance decreased (*p*_AC_ = 0.0002). Also noted was that a higher amount of CF (> 6.415%) and XG (0.31%) incorporation resulted in a pale and dark cake, respectively, leading to a decrease in color acceptance score, creating an antagonistic effect (*p*_BC_ = 0.0002) with the reduced cubic model (*p*_ABC_ = 0.0002, Figure 3O). Following the studies by Marikkar (2007), Hossain et al. (2016), and Obaroakpo, Iwanegbe, and Ojokoh (2017), we observed that substituting 6.784% RF with CF without XG (Figure 3R) resulted in the highest sensory taste acceptance (*p*_AB_ < 0.0001). Furthermore, this investigation was supported by the findings of Andrade et al. (2018) that taste acceptance decreased as the amount of XG increased (*p*_AC_ < 0.0001). Some participants disliked the strong coconut flavor from using more than 6.784% CF. Additionally, increasing the amount of XG led to a decrease in the tastiness of the cake (Figure 3T), resulting in both favorable and unfavorable outcomes at certain substitution points (*p*_BC_ < 0.0001) with a powered (lambda = 2.46) cubic model (*p*_ABC_ < 0.0001, Figure 3P). In our study, we discovered that substituting up to 9.001% of RF with CF, without XG, resulted in a sensory acceptable cake texture (*p*_AB_ < 0.0001), which was consistent with the research of Hossain et al. (2016), Marikkar (2007), and Srivastava and Semwal (2015). However, when more than 9.001% of RF was replaced with CF, the texture became inconsistent and poor, decreasing texture sensory acceptance scores (Figure 3W). Additionally, substituting XG (Figure 3Y) resulted in a rough, dry, and crusty texture, leading to lower texture sensory acceptance scores (*p*_AC_ < 0.0001), which was aligned with the findings of Marikkar (2007), Sujirtha and Mahendran (2015), and Srivastava and Semwal (2015), ultimately resulting in a reduced cubic model (*p*_ABC_ < 0.0001, Figure 3U) with an antagonistic effect of CF and XG (*p*_BC_ < 0.0001). The cake with a 5.924% substitution of RF with CF without XG received the highest overall sensory acceptance (*p*_AB_ < 0.0001). Our study also revealed diverse results (*p*_AC_ = 0.0006, Figure 3Z) regarding the impact of XG on acceptability in comparison to the findings of Chieh Sung and Chai (2017) and Andrade et al. (2018), which they reported increased overall acceptance compared to the control when incorporating 0.8%, 0.5%, and 1% xanthan gum into gluten‐free cakes based on rice‐flaxseed and fava bean flour, respectively. In the end, we observed an antagonistic effect of CF and XG (*p*_BC_ = 0.0006) through a squared root (lambda = 0.5) reduced cubic model (*p*_ABC_ = 0.0006, Figure 3V).

### 3.3 Optimized Variables Combination

**FIGURE 5:**
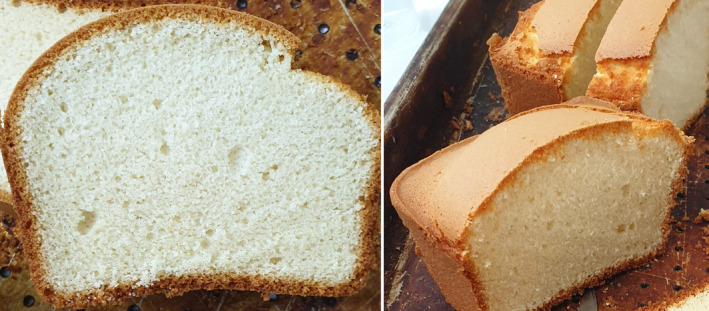
Optimized cake sample made by 23.395% rice flour, 7.795% coconut flour, and 0% xanthan gum with 0.880 desirability based on batter weight percentage.

Our research represents a significant advancement in gluten‐free product development, offering a novel approach to optimizing cake formulations using Sadri Hashemi RF, low‐fat desiccated CF, and XA. By employing a D‐optimal special cubic mixture design based on the Scheffé polynomial model, we uncovered complex, third‐degree interactions between these interdependent ingredients that were previously unexplored. This addresses a critical gap in the literature, moving beyond the commonly studied second‐degree and addition effects, and provides comprehensive models for predicting how the substitution of RF with CF and XA impacts key quality attributes in gluten‐free cakes. Unlike previous valuable studies that primarily used the OVAT method or lower‐degree models, our research developed robust models to predict both physicochemical and sensory properties at various substitution levels. The optimal formulation, based on batter weight, consisted of 23.395% RF (equal to 75.004% flour weight), 7.795% CF (equal to 24.996% flour weight), and 0% XA (Figure 5), achieving a desirability score of 0.88 (88.0%) (Figure 1). The models were validated across 11 responses within a 95% confidence interval (*p* ≤ 0.05) for upper and lower predicted intervals (PI) (Table 5), demonstrating high predictive power for batter viscosity, specific weight, Δ*E*, *a*_*w*_, moisture, texture hardness, and volume index, with pred‐*R* ^2^ values of 0.969, 0.979, 0.874, 0.931, 0.939, 0.960, and 0.985, respectively. The sensory attributes, including color, taste, texture, and overall acceptance, also exhibited strong predictive ability, with pred‐*R* ^2^ values of 0.949, 0.993, 0.996, and 0.996, respectively (Table 3). These comprehensive response models cover nearly all essential aspects of baked product quality, providing valuable insights for food producers to explore ingredient substitution and improve the quality of gluten‐free cakes while adjusting formulations based on their specific goals. This not only addresses a significant gap in gluten‐free product development but also ensures that the findings can be applied to a wide range of gluten‐free formulations, advancing the development of high‐quality gluten‐free baked goods. In summary, our research presents a novel and scientifically validated method for investigating the complex interactions of RF, CF, and XA in gluten‐free cakes. The findings provide a robust framework for optimizing gluten‐free products, addressing both technological and sensory challenges, and meeting evolving consumer needs. These contributions significantly expand the current understanding of gluten‐free baked products, providing a foundation for developing new, superior gluten‐free products.

## 4 Conclusion

This study successfully demonstrated the possibility of creating a high‐quality, gluten‐free cake that meets the sensory needs of celiac patients and those on gluten‐free diets. The research established the effectiveness of substituting RF with fiber‐rich CF, resulting in 11 significant predictive models. These models allowed for the investigation of substitution ranges up to 50% RF with CF and 1% xanthan gum, leading to a final product formulation (23.395% RF, 7.795% CF, and 0% XA) with an 88% desirability. Given the growing demand for gluten‐free products, further collaboration is recommended to develop these products, potentially through environmentally beneficial and cost‐effective by‐products. Additionally, future research should explore different rice varieties, amylose and amylopectin content, alternative gums and emulsifiers, and varied baking and mixing conditions to improve consumer acceptance.

## Author Contributions

**Aidin Taromsari:** conceptualization (lead), data curation (lead), formal analysis (lead), investigation (lead), methodology (lead), resources (lead), software (lead), validation (lead), writing – original draft (lead), writing – review and editing (equal). **Babak Ghiassi Tarzi:** methodology (supporting), resources (supporting), supervision (lead), writing – review and editing (equal).

## Ethics Statement

All panelists were briefed about the ingredients in preparation for the sensory evaluation, and written consent was subsequently obtained. As the panelists were not a vulnerable population, formal ethics approval was not required for this evaluation.

## Conflicts of Interest

The authors declare no conflicts of interest.

## Data Availability

The supporting data for this study's findings can be obtained upon reasonable request from the corresponding author. Due to privacy or ethical restrictions, the data are not available for public access.
